# Letter to the editor: is it valid to break down results from long-term trials in bipolar disorder by polarity of relapses?

**DOI:** 10.1186/s40345-014-0008-7

**Published:** 2014-06-18

**Authors:** Rasmus Wentzer Licht, Emanuel Severus

**Affiliations:** Aalborg Psychiatric University Hospital, Moelleparkvej 10, 9000 Aalborg, Denmark; Department of Psychiatry and Psychotherapy, University Hospital Carl Gustav Carus, Technische Universität Dresden, Fetscherstraße 74, Dresden, 01307 Germany

**Keywords:** Bipolar disorder, Long-term treatment, Randomised clinical trials, Methodology, Bias, Analysis, Polarity index, Mania, Depression

## Abstract

When analysing and reporting data from long-term drug trials in bipolar disorder, it has become the standard to break down the outcome into the prevention of mania and the prevention of depression. However, as illustrated by a theoretical example, this approach may confer a potential analysis bias. The point is that when mania or depression, whatever appears first, is considered an endpoint, then an endpoint of mania will exclude an endpoint of depression and vice versa. The risk of such bias is reduced when the time course is taken into consideration in the analysis.

## Correspondence

### Breaking down the analysis by polarity of relapses

When analysing and reporting data from long-term drug trials in bipolar disorder, it has become the standard to break down the outcome into the prevention of treatment-emergent mania and the prevention of treatment-emergent depression. Even such separation is usually a secondary outcome/analysis which the trials are not powered for, it plays a significant role in the interpretation of trial results, e.g. when they are qualitatively translated into guideline recommendations (Grunze et al. 2013) or quantitatively translated into a so-called polarity index (Popovic et al. 2012). The first studies where this approach revealed significant differential drug efficacies were the two pivotal studies testing lamotrigine against placebo, including lithium as a third arm to test the assay sensitivity (Bowden et al. 2003; Calabrese et al. 2003). In both studies, applying survival analysis, lamotrigine did better than placebo in terms of preventing new episodes of depression but not mania, whereas lithium prevented mania but not depression as compared to the placebo. These studies were major sources in a metanalysis by Geddes et al. (2004) addressing the preventive efficacy of lithium based on categorical data, and again, lithium did not do better than placebo in the prevention of depression. This result had a major impact on the understanding of lithium until recently when Weisler et al. (2011) in a pivotal trial on quetiapine clearly demonstrated that lithium did prevent depression better than placebo.

The breakdown-by-polarity strategy of analysis, to our knowledge, has never been questioned in the literature, although it may lead to distorted results (analysis bias). The simple point to be made here is that when mania or depression, whatever appears first, is considered an endpoint, then an endpoint of mania will exclude an endpoint of depression and vice versa. This implicates that if a drug, for example, reduces the risk of mania but not depression in comparison to placebo, then this drug will actually do worse than placebo in preventing depression, because more patients will then reach an endpoint of mania in the placebo group than in the drug group and thereby not have the chance to develop depression following mania.

### A theoretical example

Take the following theoretical extreme example: A group of 100 untreated patients with bipolar disorder are followed over a period of 2 years regardless emerging episodes. Of these, 40 patients relapse into mania after the first 0.5 year and subsequently into depression after 1.5 years, and 40 patients relapse into depression after the first 0.5 year and subsequently into mania after 1.5 years; the remaining 20 patients stay euthymic during the 2 years. Instead, we now assume that the 100 patients had been randomised into treatment with drug A, reducing the risk of mania with 50% at 0.5 year but with no influence on depression or to placebo with no preventive efficacy, and that the patients had been followed over the same specific 2 years period of time. In the drug group 10 patients would then have an endpoint of mania and 30 patients an endpoint of depression, whereas in the placebo group 20 patients would have an endpoint of mania and 20 patients would have an endpoint of depression. Therefore, the risks of reaching endpoints of mania and of depression with drug A were respectively 0.2 and 0.6 and with placebo 0.4 and 0.4, i.e. the relative risk of reaching an endpoint of depression with drug A versus placebo is 1.5. Looking at any episode as outcome, a risk of 0.8 is found in both groups.

Now moving on from the simple analysis of relative risk based on a categorical outcome measure to a Kaplan-Meier survival analysis, the probability of staying euthymic will be 0.4 after 0.5 year and 0.2 (0.4 × 0.5) after 1.5 years in the drug group and 0.2 after 0.5 year in the placebo group. The probability of not relapsing into mania (with relapses into depression censored at 0.5 year) will be 0.8 after 0.5 year and throughout in the drug group and 0.6 after 0.5 year and throughout in the placebo group. The probability of not relapsing into depression (with relapses into mania censored at 0.5 year) will be 0.6 after 0.5 year and 0.3 (0.6 × 0.5) after 1.5 year in the drug group, and 0.6 after 0.5 years and throughout in the placebo group. Here it is assumed that the only censoring taking place is that due to endpoint of the opposite polarity. Thus, there is a superior probability of staying euthymic and of not relapsing into mania in the drug group, but an inferior probability of not relapsing into depression over the 2-year study period.

Our example illustrates that not only simple analyses on categorical data in terms of computing relative risks, but also Kaplan-Meier survival analyses, may confer a potential bias when the analysis is broken down by polarity of relapse. However, the magnitude of bias is smallest with the latter approach, where the time course is taken into account, e.g. by recognising that depression after all appears relatively late in those patients who were protected from mania by the drug. Other analytical approaches like the person-year method or the Cox regression analysis will not overcome the potential bias outlined here. No matter the approach, the argument simply relies on the trivial observation that in some cases of bipolar disorder, the natural course of illness is characterised by one pole followed by the opposite pole, or vice versa (Goodwin and Jamison 1990).

## Discussion and conclusions

It can be argued that the potential bias in analysis discussed above is of minor importance under ordinary study conditions, but unfortunately, the magnitude of this bias can never be estimated empirically in real trial samples since such an estimate will require information on the clinical course of illness during the entire study period, i.e. beyond potential study endpoints under maintained/unchanged treatment conditions (placebo and/or active comparator(s)). It can also be argued that this potential bias has an only minor impact when a drug is reducing the risk of both poles equally. However, when a drug demonstrates better preventive efficacy on one pole than on the other, then it should be born in mind that such a differential efficacy may be overestimated for the reason given here, and we would warn against attempting to quantify such a differential efficacy. We would advocate for approaches that take the time course into consideration.
